# Contribution of CT Features in the Diagnosis of COVID-19

**DOI:** 10.1155/2020/1237418

**Published:** 2020-11-12

**Authors:** Houdong Zuo

**Affiliations:** Sichuan Key Laboratory of Medical Imaging, Department of Radiology, Affiliated Hospital of North Sichuan Medical College, Nanchong, Sichuan 637000, China

## Abstract

The outbreak of novel coronavirus disease 2019 (COVID-19) first occurred in Wuhan, Hubei Province, China, and spread across the country and worldwide quickly. It has been defined as a major global health emergency by the World Health Organization (WHO). As this is a novel virus, its diagnosis is crucial to clinical treatment and management. To date, real-time reverse transcription-polymerase chain reaction (RT-PCR) has been recognized as the diagnostic criterion for COVID-19. However, the results of RT-PCR can be complemented by the features obtained in chest computed tomography (CT). In this review, we aim to discuss the diagnosis and main CT features of patients with COVID-19 based on the results of the published literature, in order to enhance the understanding of COVID-19 and provide more detailed information regarding treatment.

## 1. Introduction

Coronavirus disease 2019 (COVID-19) is a highly contagious viral disease that first appeared in Wuhan, Hubei Province, China, at the end of December 2019 and rapidly spread across the country and worldwide. In January 2020, the World Health Organization (WHO) announced COVID-19 to be a major global health emergency (https://www.who.int/emergencies/diseases/novel-coronavirus-2019/situation-reports/). As of 5 July 2020, the WHO reported a total of 11,125,245 cases and 528,204 deaths due to COVID-19 (Situation report-167). At present, real-time reverse transcription-polymerase chain reaction (RT-PCR) is considered to be the standard diagnostic approach, but RT-PCR of viral nucleic acids may sometimes provide false-negative results in the initial tests [1, 2]. Therefore, some studies have reported the significance of computed tomography (CT) in diagnosing COVID-19 with higher sensitivity [3, 4]. Additionally, examination with CT has been recommended as a key tool for diagnosing and monitoring disease progression and follow-up by the National Health Commission of China (available at http://www.gov.cn/zhengce/zhengceku/2020-02/19/content_5480948.htm). The combined application of RT-PCR and CT may have advantages over single test alone and may increase the accuracy and sensitivity of diagnosis, although the algorithm of combining RT-PCR and early chest CT has not been fully studied yet and is still to be proven with more further studies [5, 6]. Different CT features have been reported by various studies at different time points, but some typical and primary characteristics can be generalized, including the bilateral and lower distribution of ground-glass opacities (GGOs), crazy-paving patterns, and consolidations in the peripheral lung area [7, 8]. With the increasing number of studies and the rise in the number of cases, more CT signs are being increasingly reported, including traction bronchiectasis, vascular enlargement, and reversed halo sign, among others [9, 10]. In order to have a better understanding of COVID-19 and achieve accurate diagnosis, as well as improve treatment and management, it is highly encouraged to focus on the diversity of COVID-19. In this review, we therefore aimed to discuss the diagnosis of COVID-19 and delineate the typical CT features based on the latest published literature, for facilitating the diagnosis and treatment of COVID-19.

## 2. The Literature Search

The COVID-19-related literature studies in this review were searched till 30 September 2020. And all the literature studies were searched based on the PubMed database (https://pubmed.ncbi.nlm.nih.gov/) using the keywords of “coronavirus,” “nCoV,” “2019-nCoV,” SARS-CoV-2, “COVID-19,” “PCR,” and “CT.” We have also included non-peer-reviewed articles from preprint repositories.

## 3. Diagnosis of COVID-19

Ever since the outbreak of COVID-19, the development of a fast and accurate diagnostic strategy has been of utmost necessity for the clinical treatment and management of the disease. RT-PCR is presently recognized as the gold standard for the diagnosis of COVID-19 [11, 12]. However, the performance of RT-PCR varies greatly, especially with respect to sensitivity [4, 13, 14]. Some new additional techniques with increased sensitivity for the diagnosis of COVID-19 were developed and demonstrated that this assay had satisfactory reproducibility in terms of cycle threshold [12, 15]. Although the sensitivity of RT-PCR has been improved greatly, the diagnostic assay still shows some false-negative results, which delays the timely treatment of patients [16]. The low or discrepant sensitivity of RT-PCR may be attributed to various reasons that are described hereafter.

First, the absence of a robust reference standard may affect the sensitivity of RT-PCR. When the combination of contact history and CT findings serve as the reference standard and is used in the COVID-19 diagnosis and results in false positives, RT-PCR sensitivity is underestimated, which means the patients without COVID-19 are diagnosed as COVID-19. On the other hand, if the RT-PCR is included in the reference standard, it may lead to “incorporation bias,” which results in overestimation of RT-PCR sensitivity in turn. The use of repeated RT-PCR testing is likely to underestimate the true rate of false negatives because not all patients in the included studies received repeated testing and those who clinically diagnosed COVID-19 were not considered to have COVID-19 actually [14, 17]. Therefore, RT-PCR with a negative result cannot exclude the possibility of COVID-19 infection and RT-PCR should not be the only criterion for diagnosis, treatment, or patient management decisions. The combination of RT-PCR and clinical features along with medical imaging evaluation facilitates the management of COVID-19 [18, 19]. Second, the sampling site and quality also affect sensitivity. In a study by Wang and coworkers [20], the sensitivity rate of RT-PCR reached 93% highest with the samples from bronchoalveolar lavage fluid (BALF), which was significantly higher than the sputum (72%), nasal swabs (63%), and fibrobronchoscope brush (46%) in COVID-19 patients. Thus, the BALF and sputum were strongly recommended for the COVID-19 diagnosis and detection of viral RNAs [19]. Third, the stage of the COVID-19, which can be interpreted as the viral load consequently, can also affect RT-PCR sensitivity. According to the natural history of the COVID-19 and viral load kinetics in different patients, sampling procedures largely contribute to the false-negative results. Optimum timing for peak viral load during infections caused by COVID-19 has great influence on the sensitivity of RT-PCR, and higher viral load may associate with higher sensitivity [17, 19]. Fourth, the results from RT-PCR using primers in different genes can be affected by viral RNA sequence variation due to the genetic diversity and rapid evolution of this novel coronavirus. False-negative results may occur by mutations in the primer and probe target regions in the novel coronavirus genome [21, 22]. The mismatches between the primers and probes and the target sequences caused by variability can result in decrease in assay performance and potential false-negative results. Finally, technical aspects of sampling may also affect the RT-PCR sensitivity, such as swab materials (should be dacron and not cotton) [23], transportation conditions, and handling of the specimens (different equipment).

Several researchers have proposed that CT scans can serve as an alternative to RT-PCR owing to better sensitivity than RT-PCR [1, 3]. Numerous studies have reported that the rate of detecting positives and the sensitivity of CT are higher than those of RT-PCR, but the observations of different studies vary owing to the differences in the inclusion criteria, reference standard, and the testing time. Consequently, studies that employ different methods produce different outcomes. The sensitivity of chest CT may be lower in confirmed cases of COVID-19 when there are fewer limitations in methodological application. Bernheim and coworkers [10] performed a retrospective study in which more than half of the CT scans were performed within 2 days after the onset of the initial symptoms. The results were proved to be normal, and only one patient with COVID-19 showed negative results in the initial RT-PCR test. This finding indicated that up to 50% of CT scans performed within 2 days of the appearance of initial symptoms were likely to be interpreted as those of a normal, uninfected individual, by an expert panel [24].

Additionally, very little information is available on the specificity of chest CT for COVID-19 owing to its limitations in distinguishing the abnormal CT findings caused by other infections [4], with those caused by COVID-19, although Bai and coworkers reported that this method has high specificity [25]. The best strategy for the diagnosis of COVID-19 appears to be the combination of CT and RT-PCR, which may render the best diagnostic efficacy [2]. He and his coworkers reported a comprehensive strategy where the initial results of RT-PCR were combined with CT. Using this strategy, an increasing number of patients with COVID-19 were correctly diagnosed, and the sensitivity of this strategy was as high as 94% (95% CI: 86–100%). The specificity and accuracy were 100% and 98%, respectively [2] (Table 1).

The discussion regarding the use of RT-PCR and CT will continue. The RT-PCR is recognized as the golden standard in the COVID-19 diagnosis, but there are false negative sometimes. CT has a relative higher sensitivity, whereas the specificity cannot be guaranteed. However, the timely application of CT in patients with COVID-19 is of utmost importance, especially in cases where the initial results of RT-PCR are negative, or in cases with no obvious symptoms but with close contact history. In addition, CT and its findings can also facilitate the stage of COVID-19 and monitor the change and evolution of this disease, which is more crucial to the option of the treatment and the management for the clinicians.

## 4. CT Features

It has been reported that pneumonia is the underlying cause of lung injury in patients with COVID-19 [28]. Therefore, the pulmonary lesions in COVID-19 are likely to be similar to those of other types of pneumonia. The majority of patients with COVID show bilateral involvement [10]. In this context, the common findings of CT can highlight the suspected or confirmed cases of COVID-19 infections. Based on the published literature on chest CT findings in relation to COVID-19, the most common features are pure GGOs, appearance of crazy-paving pattern, consolidations, thickening of interlobular septa, reticular pattern, mixed pattern, air bronchogram sign, and bronchiolectasis, among others. Furthermore, the cardinal hallmark of COVID-19 is the bilateral and lower distribution of lesions in the peripheral lung. According to previous reports, chest CT findings vary with time and disease severity [8, 10, 29]. In this review, we have delineated the common and main CT findings of COVID-19 in a pictorial fashion.

### 4.1. GGO

GGOs were first described by the Fleischner Society. GGOs are defined as blurred areas with slightly increased lung density and the absence of shading of the bronchi and edges of blood vessels, which may be attributed to the partial displacement of air resulting from partial air filling or interstitial thickening [30].

**Figure 1:**
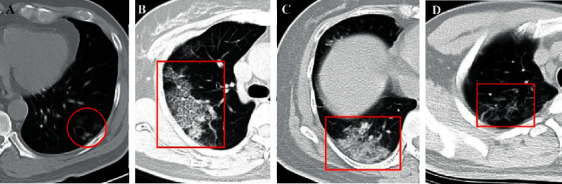
(a) A 46-year-old male COVID-19 patient with no obvious symptoms. CT scan shows three GGOs in the left lower lobe (red cycle). (b) A 46-year-old female COVID-19 patient with fever for 1 day. CT scan demonstrates crazy-paving pattern in the right upper lobe (red rectangle) (5 days after the onset of initial symptoms). (c) The same patient in (b) and the follow-up CT shows the consolidation in the right lower lobe (red rectangle) (8 days after the onset of initial symptoms). (d) A 38-year-old male COVID-19 patient with initial symptoms of dry cough and fatigue for 10+ days. CT scan shows the interlobular septal thickening in the right lower lobe (red rectangle) (7 days after the onset of initial symptoms).

Generally, single or multiple GGOs are observed in patients with COVID-19, either unilaterally or bilaterally, and are distributed peripherally in the subpleural area of the lung [29, 31, 32] (Figure 1(a)). In a study by Shi and coworkers [29], the most common pattern seen in the chest CT of 53 (65%) patients is GGO, and the presence of GGOs is likely to be the earliest CT finding in some patients, which usually appear on 0–5 days after the onset of initial symptoms [8, 33–36]. These results were consistent with those of subsequent studies on pregnant and perinatal women [37], which suggested GGO was the most common and early imaging characteristic, with the occurrence rate of up to 97.6% (81/83)–100% [9, 38, 39]. It is not yet to be understood why GGOs are the early CT manifestations, as the exact pathophysiological mechanism is poorly understood. The early pathological finding is diffuse alveolar damage, as the hyaline membrane between the alveolar walls, alveolar exudation, and edema are not obvious [34, 40]. This finding was supported by another study by Xu and coworkers, in which they performed an autopsy in a patient with COVID-19 [41]. The results demonstrated the presence of slight pulmonary edema and a hyaline membrane between the alveolar walls. Presumably, the above findings may explain the appearance of GGOs in the chest images of patients with COVID-19 (Table 2).

Pure GGOs are most common in patients with COVID-19 and those in the early stages of infection [59]. However, in an increasing number of cases, GGOs have been found to be accompanied by other signs, including reticular pattern and/or thickening of interlobular septa, appearance of crazy-paving patterns, and consolidations [8, 32, 52]. With the progression of the disease, the appearance of the typical round GGOs decreases, but the appearance of patchy GGOs and consolidations increases. We therefore hypothesize that the appearance of GGOs together with other signs may indicate disease progression and the worsening of lung injury [53].

### 4.2. Crazy-Paving Patterns

The crazy-paving pattern is another important CT feature, which shows GGOs with superimposed interlobular and intralobular septal thickening, akin to irregular paving stones [30] (Figure 1(b)), and is not as common as GGOs [42–44]. The appearance of crazy-paving patterns is frequently observed in CT findings during disease progression (5–8 days after the illness) or in severe cases of COVID-19 [8, 38, 79]. Based on the previous pathological observations for SARS-CoV, it can be hypothesized that this pattern is caused by acute and severe pulmonary injury with alveolar edema and interstitial inflammation [80]. It has been reported that the percentage of occurrence of crazy-paving patterns varies greatly. The majority of patients with COVID-19 show the appearance of crazy-paving patterns in chest images, and the rate of occurrence can be as high as 92% [44]. However, it has also been reported that this sign may not appear in the chest images of some cases of COVID-19 [47]. Although the rate of occurrence of crazy-paving patterns is relatively lower than that of GGO, it is considered to be a specific sign of COVID-19 [34] (Table 2).

### 4.3. Consolidations

Pulmonary consolidation refers to the replacement of alveolar air by pathological fluids, cells, or tissues and manifests an increase in the density of the lung parenchyma, resulting in obscuring the margins of vessels and airway walls [30], which usually is observed in the progressive or the peak stage (4–14 days after the onset of the initial symptoms) [8], but it can also be observed in the early stage (0–5 days) [36]. Angiotensin-converting enzyme is a key molecule that is involved in the development of acute lung failure [81]. Similarly, angiotensin-converting enzyme may directly induce pulmonary injury in patients with COVID-19 due to the disequilibrium of the renin-angiotensin system (RAS), which leads to diffuse alveolar damage [82]. Additionally, the appearance of consolidations may correlate with cellular fibromyxoid exudates in the alveoli [41]. These are the likely causes of the appearance of consolidations and account for the rapid changes in clinical manifestations and CT findings. Additionally, the appearance of consolidation is obvious in the progressive stages or in patient cohorts with severe COVID-19 infections [8, 29, 38]. The underlying mechanism of consolidation is in accordance with the pathological changes in the lungs of patients with COVID-19, which demonstrates all components of diffuse alveolar damage, including damage to alveolar epithelial cells, formation of a hyaline membrane, and hyperplasia of type II pneumocytes. In particular, consolidation by fibroblastic proliferation with extracellular matrix and fibrin formation are prominent among patients with COVID-19 [83].

The main CT characteristics of consolidation are multiple, patchy, or segmental regions that are distributed in the subpleural areas or along the bronchovascular bundles [9, 49, 84] (Figure 1(c)). Similar to GGO, consolidation is one of the most common CT findings among patients with COVID-19 [35, 84]. Wong and coworkers reported that consolidation is the most common CT finding (30/64, 47%), followed by GGOs (21/64, 33%), in patients with COVID-19 [84]. Additionally, consolidation can serve as an indicator of disease progression. A recent study by Zhou and coworkers [52] demonstrated that GGOs (41.9%), GGOs with reticular pattern (58.1%), and GGOs with consolidation (43.0%) are common in the early stages (1–7 days) of infection, as observed from 272 CT scans of 100 patients with COVID-19. However, the cardinal CT finding in the advanced stages (8–14 days) is GGOs with consolidation (79.8%), accompanied by repairing CT signs, including the appearance of subpleural line, distortion of the bronchus, and fibrotic strips. Another study demonstrated that pulmonary involvement gradually progressed to consolidation after 5–8 days of the initial onset of the symptoms [8]. In the progressive stage, GGOs usually evolve to consolidations and coexist with consolidations [8, 10, 52]. Additionally, the extent of consolidations is more likely to be related to the time interval between the initial onset of symptoms and the time of the CT scan and the age of the patient. Older patients with a longer time interval between the onset of symptoms and CT imaging are more likely to show consolidations [32, 52, 78] (Table 2).

### 4.4. Interlobular Septal Thickening

Interlobular septa are the 10–20 mm long linear or sheet-like structures that form the lobular borders, which are more or less perpendicular to the peripheral pleura [30]. The lobular septum consists of connective tissue, including lymphatic vessels and pulmonary veins. Patients with COVID-19 infection involving the septa may develop septal thickening, and the appearance of septal thickening is visible on chest CT images (Figure 1(d)). In thin-section CT scans, the septal thickening may appear smooth or nodular, which may facilitate the differential diagnosis of COVID-19 from other complications such as pulmonary edema that occurs in many diseases [30]. In some studies, the occurrence of interlobular septal thickening was found to be relatively high in patients with COVID-19, ranging from 0.9% to 93.6%, although it was not as common as GGOs and consolidation [4, 47, 54], and it was more likely to occur in elderly patients [57] (Table 2).

### 4.5. Air Bronchogram

**Figure 2:**
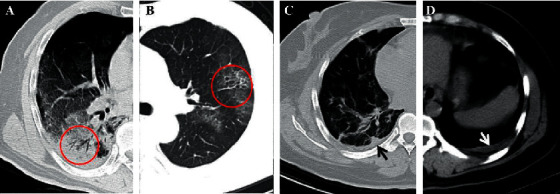
(a) A 60-year-old male COVID-19 patient with initial symptoms of dry cough and fever for 3 days. CT scan shows air bronchogram in the right lower lobe (red circle) (10 days after the onset of initial symptoms). (b) A 46-year-old male COVID-19 patient with fever. CT scan demonstrates bronchiolectasis in the left upper lobe (red circle) (14 days after the onset of initial symptoms), and the bronchial wall thickening is also observed. (c, d) A 40-year-old female confirmed patient with nasal discharge and generalized aches for 11 days. The CT shows the pleural thickening (black arrow) and a small amount of pleural effusion (white arrow) (12 days after the onset of initial symptoms).

The appearance of air bronchogram, represented by a pattern of “air-filled bronchi” with low attenuation, was observed on a background of high-density parenchyma without air [30] (Figure 2(a)). This sign is also a common CT finding recorded in a series of cases of COVID-19 [32, 48], which appears frequently in the progressive stage or the peak stage (4–14 days after the onset of the initial symptoms) [33, 53] and sometimes in the early stage [34]. However, the term of “air bronchogram” appears to be inaccurate owing to the low density of the mucus in the bronchi, which is akin to a gelatinous mucus plug rather than air. In fact, the gelatinous mucus in the bronchi may result in slight bronchial dilatation [85]. In patients with COVID-19, the gelatinous mucus plug appears to be of very low density, similar to that of air, against a background of diseased, high-density lung tissue. Ye and coworkers [85] also inferred that coughing in patients with COVID-19 could be attributed to the presence of this gelatinous mucus and damage to the dilated bronchioles (Table 3).

### 4.6. Bronchiolectasis

Bronchiolectasis is defined as the dilatation of distant bronchioles, which results from potentially reversible airway inflammation, or more frequently, retractile pulmonary fibrosis [30]. On CT images, bronchiolectasis may appear as cylindrical, varicose, or cystic, depending on the affected bronchi, but it can show a tree-in-bud pattern or appear as centrilobular nodules when the dilated bronchioles are accompanied with the thickening of the bronchial wall and mucoid impaction. In the absorption stage of the disease (usually ≥13 days), traction bronchiolectasis can be observed on the CT images, which appear as small, cystic, tubular airspaces, associated with fibrosis [30] (Figure 2(b)). Bronchiolectasis is less common in patients with COVID-19, but Dai and coworkers reported a high occurrence of up to 79% [47]. Generally, bronchiolectasis occurs in the later stages of the disease, such as in the absorptive stage, and is primarily caused by the development of fibrosis [79, 86] (Table 3).

### 4.7. Pleural Changes

The pleural changes involve pleural thickening and pleural effusion, and the former is more frequent than the latter [29, 87]. In the study by Zhou and coworkers [33], examination of the pleural changes revealed that 30 patients with COVID-19 (48.4%) showed pleural thickening in CT images, whereas only 6 patients (9.7%) had pleural effusion. Similar results were reported in a meta-analysis study with 4121 patients, in which pleural thickening (27.1%) was found to be more prevalent than pleural effusion (5.3%) [87] (Figures 2(c) and 2(d)). Based on the previous literature on Middle East respiratory syndrome coronavirus (MERS-CoV), pleural effusion may serve as a factor for poorer prognosis [38, 88]. In the study by Martino and coworkers [89] on 62 patients with COVID-19, the rate of occurrence of pleural thickening was 72.6%, which was higher than that of pleural effusion (18%). Additionally, the median value of the lung score, which was used to quantify the severity of lung involvement, was significantly higher in patients with pleural thickening than in those without this finding. Pleural effusion may serve as a potential feature for differentiating COVID-19 from other pulmonary infections, such as influenza A (H1N1) virus infections [90] (Table 2).

### 4.8. Reticular Pattern

**Figure 3:**
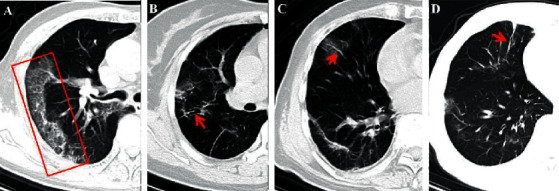
(a) A 79-year-old male COVID-19 patient with prolonged fever for 10 days. The CT displays the reticular pattern in the right upper lobe (red rectangle) (5 days after the onset of initial symptoms). (b) A 40-year-old female confirmed patient with running nose and whole-body pain for 11 days. The bronchial wall thickening is demonstrated on the follow-up CT (20 days after initial symptoms onset) (red arrow). (c) A 79-year-old male COVID-19 patient with prolonged fever for 10 days. The CT displays the vascular enlargement through the lesion in the right middle lobe (red arrow) (4 days after the onset of initial symptoms). (d) The same patient in (c) and the fibrosis is left after treatment for 10 days (red arrow).

The reticular pattern appears as a complex network of linear opacities on CT images, which is caused by interlobular and intralobular septal thickening due to the infiltration of lymphocytes [30, 41] (Figure 3(a)). In a previous study, postmortem CT revealed reticular infiltration of the lungs with severe bilateral, dense consolidation, whereas histomorphological analysis revealed diffuse alveolar damage in 8 patients [91].

Numerous studies have reported that the appearance of a reticular pattern with interlobular septal thickening is one of the most common CT findings in patients with COVID-19 and is second only to GGOs and consolidations [25, 29, 32, 69]. However, the appearance of a reticular pattern differs greatly, with the percentage of 0 to 81.8%, especially in the early stage of the disease (usually 1–7 days) [39, 52, 72]. The prevalence and frequency of reticular patterns can increase in patients with COVID-19 with disease progression [29, 92]. A study by Hu and coworkers demonstrated that the reticular patterns were obvious in 7 of 20 (45%) patients up to 14 days from the initial onset of symptoms [55]. Some studies have reported that, in some cases, reticular patterns do not appear alone, but are frequently accompanied by GGOs and other signs, including vacuolar sign, fibrotic streaks, appearance of a subpleural line, subpleural transparent line, air bronchogram, distortion of the bronchus, and pleural effusion, among others [33, 52, 60] (Table 3).

### 4.9. Thickening of the Bronchial Wall

The thickening of the bronchial wall is less common and always appears along with bronchiectasis, bronchiolectasis, and respiratory bronchiolitis-interstitial lung disease [30]. Therefore, this finding is frequently observed in critically ill patients or patients in the later stages of the disease, rather than those in the early stages [38]. Based on the literature retrieved till 17 June 2020, the highest rate of occurrence was 49.1% (55/112) [54]. The rates of occurrence reported in other studies were 1.7%–34.7% [9, 10, 38, 47, 69, 71, 74]. On CT images, the thickening of the bronchial wall shows a circular or cystic shape with thickened walls, which resembles a ring and is known as the ring sign [30] (Figure 3(b)) (Table 3).

### 4.10. Vascular Enlargement

Vascular enlargement refers to the dilatation of blood vessels around or inside the pulmonary lesions, which is always accompanied by GGOs and/or consolidation [9, 48] (Figure 3(c)). Although this sign has been rarely reported, some previous studies have reported that the incidence of this sign can be high in CT images. In a study on 143 patients with COVID-19 infection, initial abnormal chest CT revealed the occurrence of pulmonary vascular dilation in 41 of 46 patients (89.13%) in the early stage (usually 1–5 days after the onset of the symptoms) [55]. Li and Xia reported that vascular enlargement occurred in approximately 82.4% of the patients in their study [48]. Based on the reports of the published literature, vascular enlargement may be caused by alveolar and interstitial pulmonary injury and edema. The novel SARS-CoV-2 virus invades host cells through the cellular angiotensin-converting enzyme II (ACE2) receptor, and the excessive activation of immune cells leads to the production of a large quantity of inflammatory cytokines, including interleukin-6 (IL-6), which results in diffuse injury of pulmonary capillary endothelial cells and alveolar epithelium [28] (Table 3).

### 4.11. Fibrosis

Some studies have demonstrated that chest CT images of patients with COVID-19 infections show the feature of fibrosis, which is displayed in the shape of stripes, reticulations, or even honeycomb patterns [30, 31] (Figure 3(d)). The appearance of fibrosis may either indicate that the pulmonary lesions have been absorbed, or signify fibrous hyperplasia. However, evidence shows that fibrosis is more likely to develop in patients with severe infections, especially in those with high levels of inflammatory indicators, including C-reactive protein (CRP) and IL-6, and longer periods of hospitalization. During the process of COVID-19 infections, the appearance of interstitial thickening, irregular interface, coarse reticular pattern, and parenchymal band may be considered as predictors of pulmonary fibrosis, especially the appearance of an irregular interface and parenchymal band [92]. As fibrosis deteriorates with disease progression [59], patients with severe infections should be attended to more carefully, as the fibrotic changes are progressive and may result in irreversible interstitial lung disease. This may lead to the decline of pulmonary function, worsening of symptoms, poor quality of life, and early mortality [93] (Table 3). In another study by Lim and coworkers [86], as the COVID-19 improved, the resolution of a lung consolidation and development of a reticular pattern, septal thickening, and bronchiolectasis were the suggestions of fibrosis on the following second week.

At present, the relationship between fibrosis and patient prognosis is controversial. Some studies have reported that fibrosis is a reliable indicator for good prognosis, which suggests the lesions are absorbed significantly and the patient is in a stable condition [31, 35, 94]. However, some researchers support the argument that fibrosis might be an indicator of a poor prognosis because it may result in interstitial lung disease [8, 95, 96].

### 4.12. Nodules

**Figure 4:**
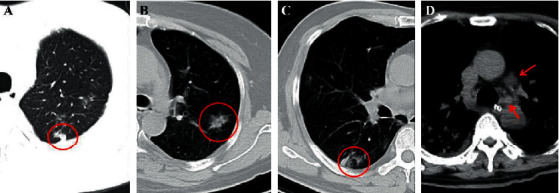
(a) A 46-year-old male COVID-19 patient with fever. CT scan demonstrates a subpleural nodule in the left upper lobe (red circle). (b) A 50-year-old female COVID-19-confirmed patient with dry cough for 4 days. The nontypical halo sign can be seen in the left upper lobe (red circle). (c) The same patient in (a) and the CT displays a nontypical reversed halo sign in the right lower lobe (red circle) in the follow-up CT image. (d) A 60-year-old male COVID-19 patient with initial symptoms of dry cough and fever for 3 days. CT scan shows the enlarged lymph nodes in the mediastinum (red arrows), and this patient is diagnosed coinfection with other bacteria.

On CT images, a nodule is defined as a round or irregular opacity which is less than 3 cm in diameter and has well or ill-defined margins [30] (Figure 4(a)). Nodules are of five types, based on the size, location, and density, and are described hereafter. Centrilobular nodules, which are located in the pleural surfaces, fissures, and interlobular septa, show soft tissue or ground-glass attenuation, with sizes ranging from a few millimeters to a centimeter. The second type of nodules is micronodules which have diameters less than 3 mm. Ground-glass nodules manifest as regions of hazy, increased attenuation in the lung, while a solid nodule appears as homogeneous soft tissue attenuation. The fifth type of nodule is the part-solid nodule or semisolid nodule, which consists of both ground-glass and solid soft tissue attenuation components [30]. The appearance of nodules is usually associated with viral pneumonia [97] and could be the initial manifestation of pneumonia caused by COVID-19 [98]. Studies have reported that multifocal solid irregular nodules were observed in 2.7–33.3% of COVID-19 patients [4, 75], while another study reported the presence of nodules with visible halo sign [55] (Table 3).

### 4.13. Halo Signs and Reversed Halo Signs

The halo sign is a terminology used in CT, which has the features of a nodule or mass surrounded by GGO [30] (Figure 4(b)). The halo sign is less common and not specific for pneumonia resulting from COVID-19, although it has been reported in several studies [34, 48, 99]. The reason underlying the appearance of this sign is unclear and could be related to angioinvasive fungal infections or hypervascular metastases that result in the development of hemorrhage around lesions, viral infections, and organizing pneumonia [30, 100, 101].

On the contrary, the reversed halo sign, which is an atoll sign, is a focal rounded GGO area surrounded by a complete or incomplete circular consolidation (Figure 4(c)). It is an uncommon sign, which was first reported specifically in cryptogenic organizing pneumonia [102], but has been subsequently associated with other diseases [103, 104]. Although numerous studies have reported this sign in patients with COVID-19, it is less common than the other signs described herein [35, 55, 74]. Li and Xia [48] reported that the reversed halo sign and pulmonary nodules with a halo sign were observed in 2 (3.9%) and 9 (17.6%) patients with COVID-19, but this sign has not been observed in patients infected with SARS-CoV or MERS-CoV. This sign may be interpreted as disease progression, which induces the formation of consolidations around GGOs or the partial absorption of lesions, leaving a low-attenuation area in the center of the lesion (Table 3).

### 4.14. Lymphadenopathy

Lymphadenopathy is not common in the COVID-19 patients. The lymphadenopathy is defined when the mediastinal lymph node short-axis diameter is more than 1 cm [30] (Figure 4(d)). This finding was reported in many studies in COVID-19 patients with the percentage of 2.7%–8% [29, 69]. In particular, lymphadenopathy was considered as a likely significant risk factor for COVID-19-patients with severe/critical pneumonia [38, 79]. However, the double or multiple bacterial infections or other viral pneumonia should be considered when lymphadenopathy sign with pleural effusion and small lung nodules are observed [3, 4, 24, 25] (Table 3).

### 4.15. Other Signs

Besides the abovementioned findings, the other uncommon or rare signs were also reported by some studies, including linear opacities [38, 78], spider web sign [38, 69], vacuolar sign [33, 52, 57], subpleural line [69, 72], and cystic change [77] (Table 3).

### 4.16. CT Findings in Children and Pregnant Women

Although COVID-19 is more common in adults, it can also occur in neonates, infants, children, and pregnant women [105–107]. In addition, some CT findings may be different from the adults. Therefore, we summarized some common CT findings of confirmed children and pregnant women, based on the published literature except the infants, because the diagnosis of neonates and infants depends on RT-PCR and related clinical data, and CT examinations cannot be performed on them.

Among children, CT findings were usually negative, and they had less extensive disease on abnormal CT scans compared with the adults [106]. The main and common in children findings were GGOs (14%–75%), consolidation (5.2%–70%), and GGO mixed with consolidation lesions (up to 36%) [99, 107–111]. The other signs, including crazy paving pattern, and the halo and reverse halo signs were also observed, which were proved to have a positive correlation between increasing age and increasing severity of findings [106]. However, Xia and coworkers [99] revealed the consolidation with surrounding halo sign was considered to be a typical sign in pediatric patients, which accounted for up to 50% cases.

In pregnant women, the most common CT finding is different. Mixed consolidation and complete consolidation were more common in the laboratory-confirmed and clinically diagnosed pregnant groups [107]. Wu and coworkers [112] found there were 65.2% of patients with patchy GGO in a single lung lobe and 34.8% patients with multiple patchy ground-glass shadows, consolidation, and fibrous stripes, and the similar result was also found in a study by Liu and coworkers [37]; they reported the most common early finding on chest CT was GGO, and with disease progression, crazy paving pattern and consolidations were seen on CT. About the different results, we infer it may be due to the different inclusion criteria, age, and the stage of the patients, which have some impact on the analysis [52, 113].

### 4.17. The Changes of the CT Findings through the Evolution of COVID-19 Infection

The temporal changes of the radiological features in relation to treatment allocated and the chronological evolution of CT features through the evolution of COVID-19 infection would be of clinical interests, which provide important information for the clinicians.

In a longitudinal study by Wang and coworkers [36], they analyzed the serial CT findings over time in 90 COVID-19 pneumonia patients. The CT scores and number of lobes involved progressed rapidly, peaked during illness days 6–11, and remained at a high level. The predominant pattern of abnormalities after symptom onset was GGO with the percentage from 45% (12–17 days) to 62% (0–5 days), and the consolidation was the second most common finding during illness days 0–5 and 6–11, with the percentage of 23% and 24%, respectively. From illness days of 0–5 to 12–17, the percentage of GGO dropped significantly from 62% to 45%, which was replaced by the significant increase of mixed pattern with the percentage of 1% to 38%, and the mixed pattern became the second most predominant pattern thereafter. The reticular pattern was rarely observed, which was presented on illness days 18–23 and ≥24 with the percentage of 3% and 6%, respectively. These findings reflected the typical pulmonary injury of viral pneumonia, which was characterized by a rapid change as reported in SARS and MERS [114, 115]. As for the temporal change of GGO, pure GGO was also the most common subtype of GGO after symptom onset. GGO with superimposed intralobular lines was the second most common subtype, with the percentage from 8% on illness days 0–5 to 28% on illness days 6–11. Of note, the percentage of GGO demonstrated a trend of “first falling then rising.” The percentage significantly dropped down from 65% on illness days 0–5 to 40% on illness days 6–11, with the increased percentages of the other 3 subtypes (GGO with interlobular thickening, GGO with intralobular lines, and irregular lies and interfaces). As the percentage significantly increased up to 71% on illness days 18–23 and thereafter, the other 3 patterns gradually decreased. The changes of these patterns probably reflect the absorption of inflammation and the reexpansion of alveoli, which also indicate the recovery of infection.

**Figure 5:**
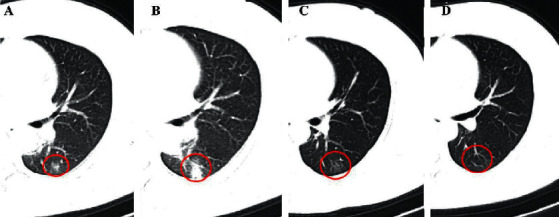
A 46-year-old female COVID-19 patient. On the initial CT (2 days after the initial symptom onset), the patchy GGO was shown in the left lower lung lobe (a) (red circle), but the lesion progressed to large opacities after approximate 5 days, with more lung tissues involvement (b). After regular treatment in hospital, the majority of the lesion was absorbed and dissipated (14 days), with little linear fibrotic lesions left (c). The lesion was absorbed completely on day 22 (d).

In another study with 21 COVID-19 patients by Pan and coworkers [8], GGO, crazy-paving pattern (GGO with superimposed inter- and intralobular septal thickening), and consolidation were the most common CT features in mild COVID-19. In most cases, the total CT score increased and peaked at 10 days after the onset of symptoms and then gradually decreased, with a total CT score of 6. Four stages were divided based on the quartiles of CT scans and degree of lung involvement from day 0 to day 26 after disease onset: stage 1 (early stage, 0–4 days), stage 2 (progressive stage, 5–8 days), stage 3 (peak stage, 9–13 days), and stage 4 (absorption stage, ≥14 days). The most common CT findings in stage 1 were GGOs with part of crazy-paving pattern and consolidation. Then the GGOs progressed to more pulmonary lobes with more crazy-paving pattern and consolidation in stage 2. In stage 3, consolidation was the main finding, with an significant decrease of GGOs and crazy-paving pattern. In the last stage, the consolidation began to alleviate and was partially absorbed without other signs. Similar results were also found in the study by Ding and coworkers [10, 78] (Figure 5).

Based on the published literature, the most common CT findings in the initial and early stage were GGO and consolidation, including patchy/punctate ground-glass opacities, and most of the GGOs progressed to multiple ground-glass infiltration in the lungs, and the consolidation became more serious in more cases in the progressive, late, or the follow-up stages. The other accompanied features included crazy-paving pattern, interstitial thickening or reticulation, air bronchograms, pleural effusion, and fibrous strips [8, 10, 31, 34, 50, 53, 55].

## 5. Conclusion

In summary, an early diagnosis and better understanding of COVID-19 infections are crucial for the treatment and management of the disease. This review comprehensively discussed the latest published literature and first-hand interpretation of CT images of pneumonia resulting from COVID-19. The review consolidates the importance of CT imaging in the diagnosis and management of COVID-19, especially in patients showing false-negative results in RT-PCR or having no obvious symptoms. Although the bilateral and peripheral distribution of GGOs and consolidations are considered to be the most common and typical imaging characteristics of patients with COVID-19 infections, the CT features may vary in different patients and at different time points. In this review, we describe the diagnosis, the typical CT features, and some uncommon CT characteristics of patients with COVID-19, for the aid of clinicians and radiologists in understanding the current diagnostic scenario and CT features for reaching a timely and accurate diagnosis. Moreover, chest CT plays an important role in follow-up, as CT features change with disease progression and expectant treatment, of which clinicians should be immediately informed.

## Figures and Tables

**Table 1:** The positive rate/sensitivity and specificity of initial RT-PCR and initial CT based on the published studies.

	Biological sample	Reference standard	Initial RT-PCR PT/Sen	Initial RT-PCR Spe	Initial CT PT/Sen	Initial CT Spe
Li et al. [14]	Pharyngeal swab	PCR(+) and CT(+)	37% (226/610)/—	—	—	—
Fang et al. [3]	Throat swab or sputum samples	PCR(+) and CT	—/71% (36/51)	—	—/98% (50/51)	—
Mardani et al. [26]	Pharyngeal swab	PCR(+)	35% (70/200)/—	—	—	—
He et al. [2]	Nasopharyngeal swab, oropharyngeal swab, endotracheal aspirate, or bronchoalveolar lavage	PCR(+) and CT(+)	—/79% (27/34)	100%((95%CI: 100%))	—/77% (26/34)	96% (95% CI: 90–100%)
Wang et al. [6]	Throat swab samples	PCR(+)	—/65% (580/888)	83% (105/126)	—/97% (580/601)	25% (105/413)
Liu et al. [13]	Nasal and pharyngeal swabs, bronchoalveolar lavage fluid, and sputum samples	PCR(+)	38% (1854/4880)/—	—	—	—
Long et al. [1]	Pharyngeal oral and nasal sampling	PCR(+)	—/83.3% (30/36)	—	—/97.2% (35/36)	—
Ai et al. [4]	Throat swab samples	PCR(+)	59% (601/1014)/—	—	88% (888/1014)/97% (580/601)	—
Guan et al. [27]	Nasal and pharyngeal swab specimens	PCR(+)	—	—	82.1% (720/877)/—	—
Bernheim et al. [10]	Bronchoalveolar lavage, endotracheal aspirate, nasopharyngeal swab, or oropharyngeal swab	PCR(+)	88% (90/102)/—	—	78% (94/121)/—	—
Bai et al. [25]	—	—	—	—	—/79.3% (Chinese); 80.5% (United States)	68.7% (Chinese); 99% (United States)

Note: all the data were obtained from the literature. The symbol “—” represents data were unavailable. The positive rate, sensitivity, and specificity are expressed as percentage. PT: positive rate; Sen: sensitivity; Spe: specificity.

**Table 2:** The most frequent CT findings acquired from the published literature.

Ref.	*N*	GGO	Crazy-paving pattern	Consolidation	GGO + consolidation	Interlobular septal thickening
Li et al. [38]	83	97.6% (81/83)	36.1% (30)	63.9% (53)	—	62.7% (52)
Guan et al. [42]	47	100% (47/47)	89.4% (42/47)	63.8% (30/47)	—	—
Miao et al. [43]	54^*∗*^	70.4% (38/54)	29.6% (16/54)	22.2% (12/54)	—	
Zhang et al. [44]	60	97% (58/60)	92% (55/60)	—	—	—
Zhang et al. [45]	34	52.9% (18/34)	23.5% (8/34)	8.8% (3/34)	35.3% (12/34)	—
Gervaise et al. [46]	72	94% (68/72)	38% (27/72)	68% (49/72)	—	—
Dai et al. [47]	219	94.5% (207/219)	—	—	—	93.6% (205/219)
Han et al. [34]	108	60.1% (65/108)	39.8% (43/108)	5.5% (6/108)	40.7% (44/108)	—
Li and Xia [48]	51	35.3% (18/51)	—	5.9% (3/51)	54.9% (28/51)	70.6% (36/51)
Bernheim et al. [10]	121	61.2% (74/121)	4.9% (6/121)			
Zhao et al. [9]	101	86.1% (87/101)	—	43.6% (44/101)	64.4% (65/101)	
Wang et al. [49]	110	27.3% (30/110)	—	27.3% (30/110)	45.4% (50/110)	—
Zhou et al. [33]	62	40.3% (25/62)	—	33.9% (21/62)	—	—
Xiong et al. [50]	42	100% (42/42)	—	81% (34/42)		69% (29/42)
Yoon et al. [51]	9 (40 lesions)	35% (14/40)	10% (4/40)	5% (2/40)	50% (20/40)	—
Cheng et al. [39]	11	100% (11/11)	—	54.5% (6/11)	63.6% (7/11)	
Zhou et al. [52]	100 (272 CT scans)	28.3% (77/272)	—	14% (38/272)	28.3% (77/272)	
Guan et al. [53]	52	96.2% (50/52)	88.5% (46/52)	78.8% (41/52)	—	
Liu et al. [54]	112	35.7% (40/112)	21.4% (24/112)	13.4% (15/112)	50.9% (57/112)	66.1% (74/112)
Hu et al. [55]	46	39.1% (18/46)	—		60.9% (28/46)	2.2% (1/46)
Li et al. [56]	131	15.3% (20/131)	—	3.1% (4/131)	46.6% (61/131)	51.9% (68/131)
Wu et al. [35]	130	53.8% (70/)	76.9% (100)	—	46.2% (60)	—
Zhu et al. [57]	72	50% (36)	—	22.2% (16)	81.9% (59)	—
Wang et al. [58]	13	30.8% (4)	—	7.7% (1)	61.5% (8)	15.4% (2)
Shang et al. [59]	307 (628 CT scans)	—	—	—	—	59.2% (372)
Vancheri et al. [60]	180	21.1% (38)	—	4.4% (8)	68.8% (124)	—
Fan et al. [61]	150	62% (93)	—	4% (6)	34% (51)	—
Luo et al. [62]	70	38.6% (27)	—	2.9% (2)	58.6% (41)	—
Chen et al. [63]	21	95.2% (20)	—	71.4% (15)	—	61.9% (13)
Cecconi et al. [64]	235	68.9% (162)	—	23.8% (56)	—	—
Iwasawa et al. [65]	6	100% (6)	100% (6)	83.3% (5/6)	—	—
Fu et al. [66]	55	32.7% (18)	16.4% (9)	14.5% (8)	52.7% (29)	38.2% (21)
Song et al. [32]	51	76.5% (39)	—	54.9% (28)	58.8% (30)	74.5% (38)
Chung et al. [67]	21	57.1% (12)	19% (4)	0 (0)	28.6% (6)	—
Pan et al. [31]	63	85.7% (54)	—	19% (12)	—	—
Xu et al. [68]	41	73.2% (30)	—	36.6% (15)	61% (25)	80.5% (33)
Wu et al. [69]	80	91.3% (73)	28.8% (23)	62.5% (50)	—	58.8% (47)
Shi et al. [29]	81	65.4% (53)	9.9% (8)	17.3% (14)	13.6% (11)	34.6% (28)
Xu et al. [70]	90	72.2% (65)	12.2% (11)	13.3% (12)	—	36.7% (33)
Liu et al. [71]	73	89% (65)	38.4% (28)	11% (8)	—	—
Zhou et al. [72]	62	61.3% (38)	25.8% (16)	1.6% (1)	35.5% (22)	—
Wang et al. [73]	1012 (917 CT scans)	94.1% (863)	—	5.9% (54)	—	—
Caruso et al. [74]	58	100% (58)	39.7% (23)	72.4% (42)	—	13.8% (8)
Himoto et al. [75]	6	66.7% (4)	0 (0)	0 (0)	33.3% (2)	—
Long et al. [1]	36	30.6% (11)	—	16.7% (6)	52.7% (19)	—
Han et al. [76]	17 (65 CT scans)	69.2% (65)	40% (26)	13.8% (9)	—	53.8% (35)
Ai et al. [4]	1014 (888 CT scans)	46.1% (409/888)	—	50.3% (447/888)	—	0.9% (8/888)
Pan et al. [8]	21 (82 CT scans)	73.2% (60)	23.2% (19)	63.4% (52)	—	—
Yang et al. [77]	149 (2376 segments)#	12.1% (287/2376)		7.2% (170/2376)	26.8% (637/2376)	—
Ding et al. [78]	112 (348 CT scans)	89.9% (313)	54.3% (189)	54.3% (189)	—	—

**Table 3:** The less frequent CT findings acquired from the published literature.

Ref.	*N*	Reticular pattern	Air bronchogram	Thickening of the bronchial wall	Bronchiolectasis	Other signs
Li et al. [38]	83	4.8% (4/83)	—	22.9% (19/83)	—	LO: 65.1% (54/83); SWS: 25.3% (21/83)
Guan et al. [42]	47	—	76.6% (36/47)	—	—	Stripe: 57.5% (27/47)
Miao et al. [43]	54^*∗*^	—	25.9% (14/54)	—	—	PT: 20.4% (11/54)
Zhang et al. [44]	60	—	93% (56/60)	—	—	—
Zhang et al. [45]	34	11.8% (4/34)	41.2% (14/34)	—	—	VE: 50% (17/34)
Gervaise et al. [46]	72	76% (55/72)	—	—	—	PE: 22% (16/72)
Dai et al. [47]	219	61.6% (135/219)	84% (184/219)	34.7% (76/219)	79% (173/219)	PT: 77.6% (170/219)
Han et al. [34]	108	—	48.1% (52/108)	—	—	VT: 79.6% (86/108); HS: 63.9% (69/108)
Li and Xia [48]	51	—	68.6% (35/51)	—	—	VE: 82.4% (42/51)
Bernheim et al. [10]	121	—	—	11.6% (12/121)	0.8% (1/121)	RHS: 1.7% (2/121)
Zhao et al. [9]	101	48.5% (49/101)	—	28.7% (29/101)	52.5% (53/101)	VE: 71.3% (72/101)
Wang et al. [49]	110	—	—	—	—	—
Zhou et al. [33]	62	62.9% (39/62)^&^	72.6% (45/62)	—	32.2% (20/62)	FS: 56.5% (35/62); VS: 54.8% (34); VE: 45.2% (28/62)
Xiong et al. [50]	42	—	62% (26/42)	—	—	FS: 74% (31/42)
Yoon et al. [51]	9 (40 lesions)	—	27.5% (11/40)	—	—	RHS: 2.5% (1/40)
Cheng et al. [39]	11	81.8% (9/11)	72.7% (8/11)	—	27.3% (3/11)	CN: 27.3% (3/11)
Zhou et al. [52]	100 (272 CT scans)	69.1% (188/272)	57.7% (157/272)	—	—	MD: 45.6% (124/272);VS: 54.8% (149/272)
Guan et al. [53]	52	—	69.2% (36/52)	—	—	IL: 71.2% (37/52)
Liu et al. [54]	112	—	19.6% (22/112)	49.1% (55/112)	—	LO: 64.2% (72/112)
Hu et al. [55]	46	—	69.6% (32/46)	—	—	VE: 89.1% (41/46); HS: 26.1% (12/46)
Li et al. [56]	131	—	57.3% (75/131)	—	—	VE: 64.1% (84/131); fibrosis: 32.8% (43/131)
Wu et al. [35]	130	—	76.9% (100/130)	—	40% (52/130)	PPS: 75.3% (98/130); VT: 76.9% (100/130); HS: 13.8% (18/130)
Zhu et al. [57]	72	61.1% (44/72)	66.7% (48/72)	—	—	VS: 50% (36/72); VE: 45.8% (33/72)
Wang et al. [58]	13	—	46.2% (6/13)	0 (0)	—	—
Shang et al. [59]	307 (628 CT scans)	—	33.6% (211/628)	—	—	VE: 66.2% (416/228)
Vancheri et al. [60]	180	15% (27/180)	—	—	—	PE: 6.6% (12/180)
Fan et al. [61]	150	—	—	—	—	—
Luo et al. [62]	70	—	—	—	—	PT: 17.1 (12/70)
Chen et al. [63]	21	—	57.1% (12/21)	—	—	VE: 66.7% (14/21)
Cecconi et al. [64]	235	—	—	—	—	—
Iwasawa et al. [65]	6	—	—	—	—	LO: 50% (3)
Fu et al. [66]	55	—	52.7% (29/55)	—	—	VT: 81.8% (45/55)
Song et al. [32]	51	21.6% (11/51)	80.3% (41/51)	—	—	PE: 7.8% (4/51)
Chung et al. [67]	21	—	—	—	—	LO: 14.3% (3)
Pan et al. [31]	63	—	—	—	—	FS: 17.5% (11/63)
Xu et al. [68]	41	—	53.7% (22/41)	—	—	TIS: 73.2% (30/41)
Wu et al. [69]	80	—	—	11.3% (9/80)	—	SWS: 25% (20/80); SL: 20% (16/80)
Shi et al. [29]	81	3.7% (3/81)	56.98% (46/81)	—	11.1% (9/81)	PT: 32.1% (26/81)
Xu et al. [70]	90	—	7.8% (7/90)	—	—	LO: 61.1% (55/90); PT: 55.6% (50/90)
Liu et al. [71]	73	—	—	26% (19/73)	—	TLT: 89% (65/73)
Zhou et al. [72]	62	0 (0)	22.6% (14/62)	0 (0)	—	RO: 25.8% (16/62); HS: 11.3% (7/62); SCL 9.7% (6/62)
Wang et al. [73]	1012	—	—	—	—	—
Caruso et al. [74]	58	—	36.2% (21/58)	1.7% (1/58)	41.4% (24/58)	HS: 12.1% (7/68);
Himoto et al. [75]	6	—	—	0 (0)	—	PN: 33.3% (2/6)
Long et al. [1]	36	—	—	—	—	LYM: 2.8% (1/36); PE 5.6% (2/36)
Han et al. [76]	17 (65 CT scans)	0 (0)	41.5% (27/65)	—	3.1% (2/65)	VE: 64.6% (42/65)
Ai et al. [4]	1014 (888 CT scans)	—	—	—	—	NL: 2.7% (24/888)
Pan et al. [8]	21 (82 CT scans)	—	—	—	—	—
Yang et al. [77]	149 (2376 segments)^#^	53% (79/149)	54.4% (81/149)	—	17.4% (26/149)	SLO: 20.8% (31/149); CC: 8.1% (12/149); PE: 6.7% (10/149)
Ding et al. [78]	112 (348 CT scans)	—	36.5% (127/348)	—	25.6% (89/348)	LO: 54% (188/348); PE 17% (59/348)

Note: GGO: ground-glass opacity. The symbol “*∗*” represents the total number of patients with positive RT-PCR. Symbol “&” represents reticular pattern and GGO. Symbol “#”: 149 is the total number of COVID-19 patients. The number in the parentheses represents the total of pulmonary segments in 132 patients with abnormal CT findings (132 × 18 = 2376, 18 pulmonary segments/1 patients). Some percentages are calculated based on the total segments. “—”: not available. LO: linear opacities; SWS: spider web sign; PT: pleural thickening; VE: vascular enlargement; VT: vascular thickening; HS: halo sign; RHS: reverse halo sign; FS: fibrous stripes; VS: vacuolar sign; CN: centrilobular nodules; MD: microvascular dilation; IL: irregular line; PPS: parallel pleura sign; VES: vascular enhancement sign; PE: pleural effusion; SL: subpleural line; TIS: thickened intralobular septa; TLT: thickening of lung texture; RO: rounded opacities; SCL: subpleural curvilinear line; PN: pulmonary nodules; LYM: Lymphadenopathy; NL: nodular lesions; SLO: subpleural linear opacity; CC: cystic change.

## Abbreviations

SARS-CoV-2: Severe acute respiratory syndrome coronavirus 2
MERS: Middle East respiratory syndrome.
